# Induction of Premature Labor

**Published:** 1868-07

**Authors:** 


### Induction of Premature Labor.

Undoubtedly the best way of inducing premature labor is that known as Cohen's. In this plan fluid is injected between the uterine walls and the membranes by means of a catheter. A great improvement on this is to use a tube with a single aperture at the end instead of with side apertures. The object of this is that the fluid may pass directly upwards to the fundus of the uterus. Experience shows that for sure action of injection, it is necessary that the fluid injected should approach as near as possible to the fundus of the uterus, this being the most sensitive to irritation. (Prof. J. Lazarewitch.)

The following is the mode of application of the douche for the purpose of inducing premature labor. Place the patient in the usual obstetric position, with the hips drawn well over the edge of the bed; by passing a full sized Ferguson's speculum the os may then be brought into view and the nozzle of an ordinary syphon syringe inserted into it. A continuous stream of water must then be injected into the cavity of the uterus. Before using the syringe care must be taken to fill it completely with water, so as to exclude the admission of any air into the uterine sinuses. Tepid water answers very well, and it is unnecessary to use alternately hot and cold water. (Dr. T. Telford.)—*Braithwaite's Retrospect.*

The above method is dangerous, and is the very last to be practiced. Several deaths have been caused by it, and though it has some advantages, still the liability of air being conveyed into the uterine sinuses and thus into the circulation, should cause this method to be abandoned, or adopted under the most careful supervision and from great necessity; indeed we would never adopt or recommend it.—Ed.

